# Prophylactic Platelets in Dengue: Survey Responses Highlight Lack of an Evidence Base

**DOI:** 10.1371/journal.pntd.0001716

**Published:** 2012-06-26

**Authors:** James Whitehorn, Rosmari Rodriguez Roche, Maria G. Guzman, Eric Martinez, Wilmar Villamil Gomez, Leonard Nainggolan, Ida Safitri Laksono, Ajay Mishra, Lucy Lum, Abul Faiz, Amadou Sall, Joshua Dawurung, Alvaro Borges, Yee-Sin Leo, Lucille Blumberg, Daniel G. Bausch, Axel Kroeger, Olaf Horstick, Guy Thwaites, Heiman Wertheim, Mattias Larsson, Tran Tinh Hien, Rosanna Peeling, Bridget Wills, Cameron Simmons, Jeremy Farrar

**Affiliations:** 1 Department of Clinical Research, London School of Hygiene and Tropical Medicine, London, United Kingdom; 2 Hospital for Tropical Diseases, Oxford University Clinical Research Unit, Wellcome Trust Major Overseas Programme, Ho Chi Minh City, Vietnam; 3 Instituto de Medicina Tropical Pedro Kouri, Havana, Cuba; 4 Hospital Universitario de Sincelejo, Sincelejo, Colombia; 5 Faculty of Medicine, University of Indonesia, Jakarta, Indonesia; 6 Paediatric Department, Gadjah Mada University, Yogyakarta, Indonesia; 7 Sunderlal Memorial Hospital, Delhi, India; 8 Department of Paediatrics, Faculty of Medicine, University of Malaya, Kuala Lumpur, Malaysia; 9 Sir Sallimullah Medical College, Dhaka, Bangladesh; 10 Institute Pasteur, Dakar, Senegal; 11 University of Maiduguri Teaching Hospital, Maiduguri, Borno State, Nigeria; 12 Copenhagen HIV Programme, University of Copenhagen, Faculty of Health Sciences, Copenhagen, Denmark; 13 University Hospital, Federal University of Minas Gerais, Belo Horizonte, Brazil; 14 Department of Infectious Diseases, Tan Tock Seng Hospital, Singapore, SIngapore; 15 National Institute for Communicable Diseases, Johannesburg, South Africa; 16 Tulane School of Public Health and Tropical Medicine, New Orleans, Louisiana, United States of America; 17 Special Programme for Research and Training in Tropical Diseases, World Health Organization, Geneva, Switzerland; 18 Institute of Public Health, University of Heidelberg, Heidelberg, Germany; 19 Department of Infectious Disease/Centre for Clinical Infection and Diagnostics Research, King's College London, London, United Kingdom; Pediatric Dengue Vaccine Initiative, United States of America

## Abstract

Dengue is the most important arboviral infection of humans. Thrombocytopenia is frequently observed in the course of infection and haemorrhage may occur in severe disease. The degree of thrombocytopenia correlates with the severity of infection, and may contribute to the risk of haemorrhage. As a result of this prophylactic platelet transfusions are sometimes advocated for the prevention of haemorrhage. There is currently no evidence to support this practice, and platelet transfusions are costly and sometimes harmful. We conducted a global survey to assess the different approaches to the use of platelets in dengue. Respondents were all physicians involved with the treatment of patients with dengue. Respondents were asked that their answers reflected what they would do if they were the treating physician. We received responses from 306 physicians from 20 different countries. The heterogeneity of the responses highlights the variation in clinical practice and lack of an evidence base in this area and underscores the importance of prospective clinical trials to address this key question in the clinical management of patients with dengue.

## Author Summary

A low platelet count is a common feature of dengue infection. It is thought that the platelet count correlates with the severity of the infection and may contribute to the risk of developing haemorrhage, a well-recognised complication of dengue. As a result of this platelet transfusions are used in some settings to reduce the risk of haemorrhage. There is currently no evidence to support this practice, and platelet transfusions are costly and sometimes harmful. We conducted a survey assessing the use of platelets in dengue. Respondents were all physicians involved with the treatment of patients with dengue. Respondents were asked that their answers reflected what they would do if they were the treating physician. We received 306 responses from 20 different countries. The striking feature of the survey responses was the heterogeneity of approaches to the use of platelets in dengue. These findings highlight the variation in clinical practice and lack of an evidence base in this area and underscore the importance of conducting prospective clinical trials to address this key question in dengue clinical management.

## Introduction

Dengue is globally the most important arboviral infection and threatens an estimated 2.5 billion people worldwide [1]. Thrombocytopenia is almost universally observed in dengue infection [2]. This results from both reduced production and increased destruction of platelets [3]–[5]. It is thought that severe thrombocytopenia correlates with disease severity and may contribute to the risk of developing haemorrhage [6], [7]. The 2009 WHO dengue guidelines do not advocate the use of prophylactic platelet transfusions, whereas the 2011 regional WHO guidelines for South East Asia suggest prophylactic platelets may be considered in those with a platelet count less than 10×10^9^/L [8], [9]. Some dengue-endemic countries support the use of prophylactic platelet transfusions to prevent haemorrhage in patients with thrombocytopenia, for example India (<10×10^9^/L), whereas others, such as Brazil, do not [10], [11]. However platelet transfusions are costly, potentially dangerous and their use in dengue lacks an evidence base [12]–[15].

## Methods

We conducted a survey among physicians directly involved in the care of dengue patients in order to determine how platelets are used in the clinical management of dengue. The majority of respondents were practicing physicians in dengue-endemic areas. The exceptions to this were respondents from Africa, where dengue is emerging, and the UK where the respondents were infectious disease physicians who regularly see patients who have recently travelled to dengue-endemic areas. A questionnaire containing nine case histories and an additional question about prophylactic platelet transfusion thresholds was emailed to physicians with experience in managing dengue patients and known to us. Respondents were specifically asked that their responses reflect what they would do if they were the treating physician. Email recipients were invited to further disseminate the questionnaire within their own clinical networks. The complete list of questions is available as a supplementary file (Questionnaire S1).

The case histories were based on real clinical cases seen at the Hospital for Tropical Diseases in Ho Chi Minh City, Vietnam. Four case histories describe patients with clinically non-severe dengue but varying levels of thrombocytopenia. Case 1 describes an 18-year-old female with platelets of 23×10^9^/L and no bleeding. Case 2 describes a 28-year-old male with platelets of 29×10^9^/L. He had no bleeding but a past history of a perforated peptic ulcer. Case 3 describes a 29-year-old female with a rapid fall in platelets to 22×10^9^/L. She had no bleeding. Case 4 describes a 30-year-old male with platelets of 3×10^9^/L and no bleeding. Five case histories describe patients with different manifestations of severe dengue associated with varying levels of thrombocytopenia. Case 5 describes a 19-year-old male with platelets of 18×10^9^/L. He had dengue hepatitis but no bleeding. Case 6 describes a 20-year-old female with platelets of 17×10^9^/L. She had suspected dengue encephalitis but no bleeding. Case 7 describes a 24-year-old male with platelets of 31×10^9^/L. He had hepatic failure thought to be secondary to dengue but no bleeding. Case 8 describes a 23-year-old female with platelets of 8×10^9^/L. She had shock, epistaxis and vaginal bleeding. Case 9 describes a 23-year-old male with platelets of 33×10^9^/L. He had shock and mucosal bleeding. The final question aimed to determine thresholds at which a physician would consider transfusing platelets as prophylaxis against haemorrhage. Respondents were asked to select a single option.

## Results

In total, 306 physicians from 20 different countries responded within a specified time period. The responses from Asia were 52 from Indonesia, 7 from Bangladesh, 5 from the Philippines, 9 from Singapore, 8 from Cambodia, 18 from Malaysia, 3 from Thailand, 20 from Vietnam and 12 from India. The responses from Latin America and the Caribbean were 13 from Cuba, 10 from Brazil, 17 from Paraguay, 6 from Peru, 1 from Mexico, 1 from Bolivia, 1 from Martinique and 81 from Colombia. The responses from Africa were 37 from Nigeria and 2 from South Africa. In addition there were 3 responses from the UK.

Among the 4 case histories describing patients with clinically non-severe dengue associated with varying levels of thrombocytopenia, 16–24% of respondents recommended platelet transfusion at platelet concentrations of 22–29×10^9^/L., but approximately one-third of the respondents would transfuse platelets if the count fell to 3×10^9^/L. (Table 1)

**Table 1:** Proportion of respondents choosing to transfuse platelets stratified by geographic region (n, (%)); BP = blood pressure; HR = heart rate; HCT = haematocrit.

Clinical case	Asia (n = 134)	Africa (n = 39)	S. America & Caribbean (n = 130)	UK (n = 3)	Total (n = 306)
**Case 1** 18-year-old female; platelets 23×10^9^/L; no haemorrhage; BP 120/80; HR 105; HCT 39%	12 (9)	38 (97.4)	8 (6.2)	0 (0)	58 (19)
**Case 2** 28-year-old male; platelets 29×10^9^/L; no haemorrhage; past history of perforated gastric ulcer; BP 100/75; HR 92; HCT 42%3	20 (14.9)	39 (100)	13 (10)	0 (0)	72 (23.5)
**Case 3** 29-year-old female; rapid fall in platelets to 22×10^9^/L; no haemorrhage; haemodynamically stable	12 (9)	30 (76.9)	6 (4.6)	0 (0)	48 (15.7)
**Case 4** 30-year-old male; platelets 3×10^9^/L; no haemorrhage; haemodynamically stable	57 (42.5)	37 (94.9)	23 (17.7)	3 (100)	120 (39.2)
**Case 5** 19-year-old male; platelets 18×10^9^/L; dengue hepatitis; no haemorrhage; BP 90/60; HR 120; HCT 47%	11 (8.2)	36 (92.3)	7 (5.4)	2 (66.7)	56 (18.3)
**Case 6** 20-year-old female; platelets 17×10^9^/L; dengue encephalitis; no haemorrhage; BP 100/70; HR 100; HCT 40%	25 (18.7)	39 (100)	19 (14.6)	1 (33.3)	84 (27.5)
**Case 7** 24-year-old male; platelets 31×10^9^/L; hepatic failure secondary to dengue; no haemorrhage; BP 125/70; HR 110; HCT 42%	5 (3.7)	39 (100)	9 (6.9)	0 (0)	53 (17.3)
**Case 8** 23-year-old female; platelets 8×10^9^/L; shock, epistaxis and vaginal bleeding; BP 75/50; HR 110; HCT 42%	86 (64.2)	39 (100)	43 (33.1)	3 (100)	171 (55.9)
**Case 9** 23-year-old male; platelets 33×10^9^/L; shock and mucosal bleeding; BP 70/50; HR 120; HCT 46%	57 (42.5)	39 (100)	26 (20)	1 (33.3)	123 (40.2)
**Prophylactic platelet transfusion threshold:**					
<50×10^9^/L	8 (6)	23 (59)	0 (0)	0 (0)	31 (10.1)
<40×10^9^/L	1 (0.7)	7 (17.9)	0 (0)	0 (0)	8 (2.6)
<30×10^9^/L	1 (0.7)	7 (17.9)	2 (1.5)	0 (0)	10 (3.3)
<20×10^9^/L	12 (9)	1 (2.6)	2 (1.5)	2 (66.7)	17 (5.6)
<10×10^9^/L	33 (24.6)	0 (0)	12 (9.2)	1 (33.3)	46 (15)
Not in absence of bleeding	75 (56)	1 (2.6)	39 (30)	0 (0)	190 (62.1)

Among the 5 case histories describing patients with different manifestations of severe dengue associated with varying levels of thrombocytopenia, more respondents would transfuse platelets if the patient was in shock and bleeding (case histories 8 and 9). There were substantial differences in the responses from physicians in Africa than those from Asia and America for all 9 cases (Table 1).

The final question aimed to determine thresholds at which a physician would consider transfusing platelets as prophylaxis against haemorrhage. Respondents were asked to select a single option. 31 (10%) respondents would consider a prophylactic platelet transfusion if the platelet count was below 50×10^9^/L. 8 (2.6%) respondents would consider a prophylactic platelet transfusion if the platelet count was below 40×10^9^. 10 (3.3%) respondents would consider a prophylactic platelet transfusion if the platelet count was below 30×10^9^. 17 (5.6%) respondents would consider a prophylactic platelet transfusion if the platelet count was below 20×10^9^. 46 (15%) respondents would consider a prophylactic platelet transfusion if the platelet count was below 10×10^9^. 190 (62%) respondents would only consider transfusing platelets in patients with signs of haemorrhage.

The responses categorised by global region are summarised in Table 1.

## Discussion

Our study has limitations. There is an element of selection bias in the way the survey was conducted, as the physicians who distributed the survey within their countries were known to have an interest in dengue. The survey is subject to response bias meaning that the answers may not accurately reflect clinical practice in the respective countries. In addition, the country representation is not balanced.

Despite these limitations the striking result of this survey is the heterogeneity of approaches to the use of prophylactic platelet transfusions in dengue. 112/306 respondents would consider transfusing platelets prophylactically at various levels of thrombocytopenia. When the responses are categorised by region (Table 1) African respondents would advocate platelet transfusions more frequently, perhaps reflecting more limited experience with dengue and experience with other haemorrhagic fevers. The choice to use prophylactic platelet transfusions may be influenced by cost and availability of platelets, as well as individual experience in managing dengue and other medical conditions that affect the platelet count. There is considerable variability within countries suggesting an individual's practice may differ from recommendations in guidelines. For example 6/12 Indian respondents and 7/10 Brazilian respondents would consider the use of prophylactic platelets. The responses reflect wide variation in clinical practice and are indicative of the paucity of clinical evidence to guide practice in this area.

At present there is limited evidence to support the use of prophylactic platelet transfusions in dengue despite their inclusion in some national guidelines. As the global reach of dengue continues to expand the need to conduct clinical trials to construct an evidence base to guide the appropriate use of platelets in dengue becomes ever more pressing.

## Supporting Information

Questionnaire S1 **Dengue clinical scenarios.**
(DOC)
