# Supplementary material for: Prophylactic Platelets in Dengue: Survey Responses Highlight Lack of an Evidence Base
Source: PLoS Negl Trop Dis. 2012 Jun 26;6(6):e1716. doi: 10.1371/journal.pntd.0001716 (PMC3383756; doi:10.1371/journal.pntd.0001716)
Supplement: Questionnaire S1 — Dengue clinical scenarios. (DOC) [file pntd.0001716.s001.doc]

**Dengue clinical scenarios**

Case 1:

A 18-year-old woman is seen in the emergency clinic. She has been unwell for 5 days with a high fever, headache and has developed a rash. On examination she is febrile (38.4), her pulse is 105 and her blood pressure is 120/80. Her rapid diagnostic test for dengue is positive. Her other investigations are as follows:

| HB = 14.2g/dL | AST = 65 IU/L |
| --- | --- |
| WCC = 3.4 x10^9^/L | ALT = 50 IU/L |
| PLT = 23 x10^9^/L | Clotting profile normal |
| Hematocrit = 39% |  |

Which of the following would be part of your management plan (tick all that apply)?

- Discharge home with advice and anti-pyretics Ω
- Admission for observation Ω
- Platelet transfusion Ω
- Parenteral fluids Ω

Case 2:

A 28-year-old man is admitted to the ward with dengue (confirmed serologically). He has had a fever for 7 days. 6 months ago he was admitted to hospital with a severe upper gastrointestinal haemorrhage secondary to a perforated peptic ulcer. Currently he is febrile (38.2), his BP is 100/75 and his pulse is 92. Investigations are as follows:

| HB = 15.2g/dL | AST = 62 IU/L |
| --- | --- |
| WCC = 5.1 x10^9^/L | ALT = 45 IU/L |
| PLT = 29 x10^9^/L | APTT = 45 seconds PT = 12 seconds |
| Hematocrit = 42% |  |

Which of the following would be part of your management plan (tick all that apply)?

- Parenteral fluids Ω
- Platelets to prevent haemorrhage (given history of peptic ulceration) Ω
- Upper GI endoscopy Ω
- Chest x-ray and ultrasound Ω

Case 3:

A 29-year-old female with confirmed dengue (NS1 and PCR positive) has been admitted for observation on the 3rd day of illness. On the second day of admission she remains febrile (38.8) but is otherwise stable. However the laboratory calls you to inform you that her platelet count has fallen from 102 x10^9^/L to 22 x10^9^/L. You re-examine her with this result in mind but find no evidence of haemorrhage. Her other blood tests, including hematocrit and a clotting profile are normal.

Which of the following would you do (tick all that apply)?

- Discharge home with advice to return if complications develop Ω
- Continue to observe Ω
- Administer platelets and observe as an inpatient Ω
- Arrange imaging to look for evidence of occult haemorrhage Ω

Case 4:

A 30-year-old man is seen in the clinic with suspected dengue. He has a 5-day history of fever, malaise and headache. On examination he is febrile (38.8) and has a rash that is suggestive of dengue. He is haemodynamically stable and has no evidence of haemorrhage. The haematology laboratory phone you to inform you that his platelet count is 3 x10^9^/L. Would you consider arranging a platelet transfusion?

- Yes Ω
- No Ω

Case 5:

A 19-year-old man with suspected dengue is admitted on the 6th day of illness. He has a rash suggestive of dengue infection and significant bruising at venepuncture sites. He denies any bleeding. He is afebrile (36.2), his blood pressure is 90/60 and his pulse is 120. Investigations are as follows:

| HB = 15.2g/dL | AST = 320 IU/L |
| --- | --- |
| WCC = 2.1 x10^9^/L | ALT = 160 IU/L |
| PLT = 18 x10^9^/L | APTT = 49 seconds PT = 13 seconds |
| Hematocrit = 47% |  |

Which of the following would be part of your management plan (tick all that apply)?

- Administer parenteral fluids Ω
- Admit to a high dependency area for careful monitoring Ω
- Ultrasound of liver Ω
- Platelet transfusion to prevent haemorrhage Ω

Case 6:

A 20-year-old woman is admitted with fever and a reduced level of consciousness. According to her family she has been unwell for 4 days complaining of a headache in addition to her fever. The day prior to admission she had an episode of vomiting. On examination she has a reduced GCS (11/15), she is febrile (38.5), her pulse is 100 and her blood pressure is 100/70. She has scattered petechiae on her lower limbs but no other evidence of bleeding. She has a CT scan of her brain, which is normal, a blood film for malaria, which is negative and a lumbar puncture that shows 92 white cells (90% lymphocytes) with normal biochemistry. She has a positive NS1 rapid test for dengue and you suspect she may have dengue encephalitis. Her other investigations are as follows:

| HB = 12.9 g/dL | AST = 165 IU/L |
| --- | --- |
| WCC = 2.9 x10^9^/L | ALT = 135 IU/L |
| PLT = 17 x10^9^/L | Clotting profile normal |
| HCT = 40% |  |

Which of the following form part of your management plan (tick all that apply)?

- Admit for neurological observation Ω
- Treat with parenteral aciclovir while awaiting CSF PCR result Ω
- Administer platelets as prophylaxis against haemorrhage Ω
- Commence parenteral fluids Ω

Case 7:

A 24-year-old man with suspected dengue is admitted to hospital. He has had a fever for 5 days. On examination he is febrile (38.6), his pulse is 110 and his blood pressure is 125/70. Physical examination reveals some mild bruising and mild hepatomegaly with tenderness on palpation of his right upper quadrant. Dengue is confirmed by PCR. Investigations are as follows:

| HB = 13.6 g/dL | AST = 1845 IU/L |
| --- | --- |
| WCC = 4.9 x10^9^/L | ALT = 1250 IU/L |
| PLT = 31 x10^9^/L | APTT = 72 seconds PT = 19 seconds |
| HCT = 42% |  |

Which of the following would be part of your management plan (tick all that apply)?

- Admission for observation Ω
- Parenteral fluids Ω
- Institute supportive treatment for pending hepatic failure Ω
- Platelets Ω
- Vitamin K Ω

Case 8:

A 23-year-old woman is admitted on the 7th day of illness. She has dengue as confirmed by an NS1 assay. She reports some epistaxis and abnormal menstrual bleeding. On examination she has scattered petechiae and bruising at venepuncture sites. She has a temperature of 37.9, her blood pressure is 75/50 and her pulse is 110. Investigations are as follows:

| HB = 8.9g/dL | AST = 125 IU/L |
| --- | --- |
| WCC = 9.2 x10^9^/L | ALT = 107 IU/L |
| PLT = 8 x x10^9^/L | APTT = 61 seconds PT = 17 seconds |
| Hematocrit = 42% |  |

Which of the following would be part of your management plan (tick all that apply)?

- Administer parenteral fluids Ω
- Admit to HDU Ω
- Administer packed red cells Ω
- Administer platelets Ω
- Start IV heparin Ω
- Chest x-ray and ultrasound Ω
- Nasal packing Ω

Case 9:

A 23-year-old man is referred to the hospital with confirmed dengue (NS1 positive). He has been unwell for a week. He had been treated with parenteral fluids at the referring health centre. On examination he is afebrile, his pulse is 120 and his blood pressure is 70/50. There is widespread bruising at the site of venepuncture as well as scattered petechiae elsewhere. On examination of his mouth there is evidence of some mucosal bleeding. While examining him he has haemetemesis. His investigations are as follows:

| HB = 10.1 g/dL | AST = 324 IU/L |
| --- | --- |
| WCC = 7.2 x10^9^/L | ALT = 127 IU/L |
| PLT = 33 x10^9^/L | APTT = 68 seconds; PT = 16 seconds |
| HCT = 46% |  |

Which of the following would form part of your management plan (tick all that apply)?

- Parenteral fluids Ω
- Packed red cells Ω
- Platelets Ω
- Insertion of CVP line Ω
- Admission to intensive care facility Ω

Question 10:

At what level would you consider prescribing platelets as prophylaxis against haemorrhage (please chose 1 response)?

- <50 x10^9^/L Ω
- <40 x10^9^/L Ω
- <30 x10^9^/L Ω
- <20 x10^9^/L Ω
- <10 x10^9^/L Ω
- In the absence of haemorrhage I would not prescribe platelets Ω
